# Autoimmune Polyglandular Syndrome II: A Case Report

**DOI:** 10.7759/cureus.52372

**Published:** 2024-01-16

**Authors:** Olfat Awad, Hadil Basma, Rim Masri, Samih Hamadeh, Majdi Hamadeh

**Affiliations:** 1 Department of Nephrology, Lebanese University Faculty of Medical Sciences, Beirut, LBN; 2 Department of Endocrinology, Lebanese University Faculty of Medical Sciences, Beirut, LBN; 3 Department of Internal Medicine, Wayne State University Detroit Medical Center, Detroit, USA; 4 Department of Nephrology, Al-Zahraa Hospital University Medical Center, Beirut, LBN

**Keywords:** siadh, hyponatremia, case report, aps ii, adrenal insufficiency, diabetes

## Abstract

Autoimmune polyglandular syndrome II (APS-II), also known as Schmidt syndrome, is a rare endocrine disorder characterized by endocrine and non-endocrine illnesses. Addison’s disease and at least one additional autoimmune condition, such as autoimmune thyroid disease or type 1 diabetes mellitus (T1DM), are features of APS-II. It can result from genetic and non-genetic factors. We present a case of a 60-year-old female patient with a history of T1DM and a recent diagnosis of Hashimoto's thyroiditis who was admitted to the nephrology department for hyponatremia. Investigations showed the presence of adrenal insufficiency (AI), so she was diagnosed with APS-II and had the full triad of this syndrome. Thus, it is important to think about the diagnosis of AI or other autoimmune conditions in a patient who already has one or more autoimmune diseases.

## Introduction

Autoimmune polyglandular syndromes (APSs) are uncommon polyendocrinopathies that are defined by the dysfunction of multiple endocrine organs due to autoimmune processes with a high risk of non-glandular manifestations. APSs are due to genetic and non-genetic environmental factors. They are divided into juvenile (APS-I) and adult types; the latter is subdivided into three subtypes (II-IV) based on clinical features and mode of inheritance [1,2].

Primary adrenal insufficiency (AI), autoimmune thyroid disease (ATD), and type 1 diabetes mellitus (T1DM) are components of APS-II that were initially described by Schmidt in 1926 [1]. It is a polygenetic inherited disease with multiple mutations identified, whereas APS-I has monogenic inheritance. The prevalence of APS-II is 1:20,000 [2]. Women are more frequently affected than men, with a ratio of 1:3. It generally occurs in the third and fourth decade, with peak incidence at ages 20-60 years [3]. Given the significant associated morbidity and potential mortality of autoimmune polyglandular diseases, there should be a high index of suspicion to diagnose APS-II as early as possible to avoid complications and ameliorate patient prognosis [3]. We report a case of a 60-year-old female patient presenting with a complete triad of APS-II.

## Case presentation

We present a case of a 60-year-old female patient known to have had T1DM for 20 years maintained on insulin therapy, osteoarthritis, and controlled epilepsy on lamotrigine. A recent blood workup revealed an elevated thyroid-stimulating hormone (TSH) level at 6 mU/ml (0.27-4.2) and an elevated anti-thyroid peroxidase (anti-TPO) titer at 251 IU/ml (<34 IU/ml) with normal free T3 (FT3) and free T4 (FT4) levels. Based on this, she was also diagnosed with subclinical hypothyroidism.

She was a non-smoker, non-alcoholic, and had no significant family history. She had no history of pre-menopausal menstrual irregularities or galactorrhea.

Before the patient’s presentation to our emergency department, she was in another hospital receiving a hyaluronic acid injection in her knee to control pain-induced osteoarthritis, and laboratory tests over there showed hyponatremia of 126 mmol/l (135-145), so she was sent to our hospital for more investigations. The patient mentioned having fatigue and generalized weakness. There were no other associated symptoms.

**Figure 1:**
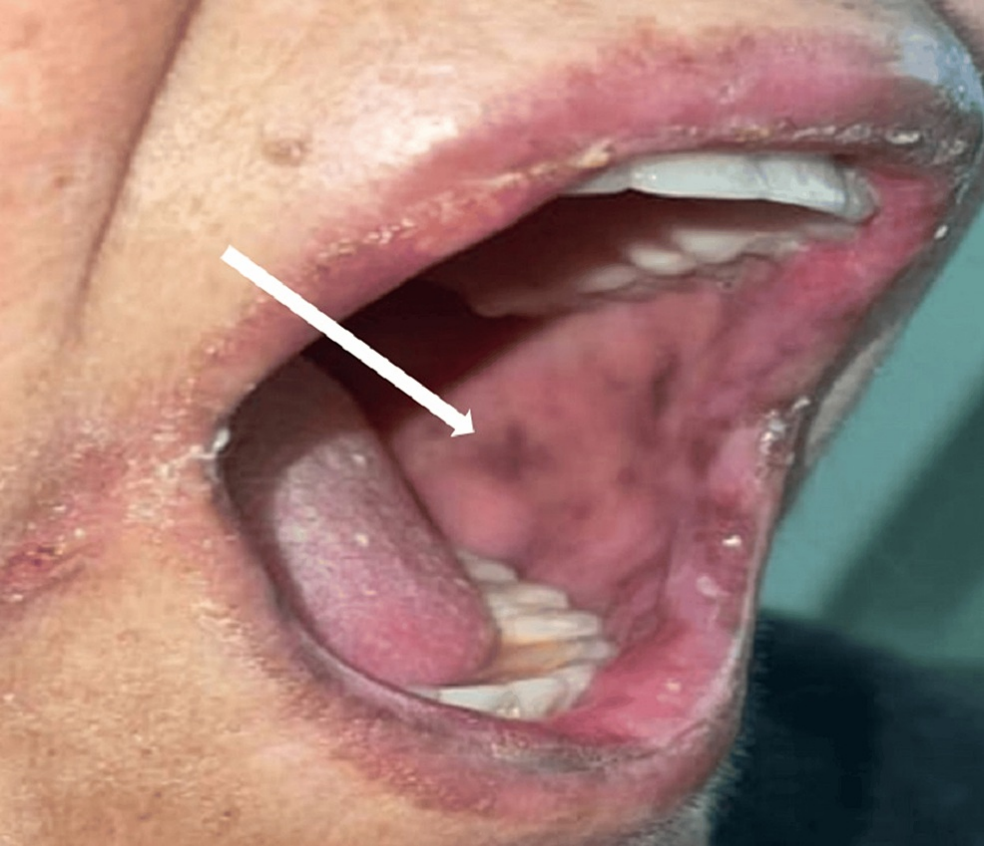
Mucosal hyperpigmentation in the oral cavity (marked by the white arrow).

**Figure 2:**
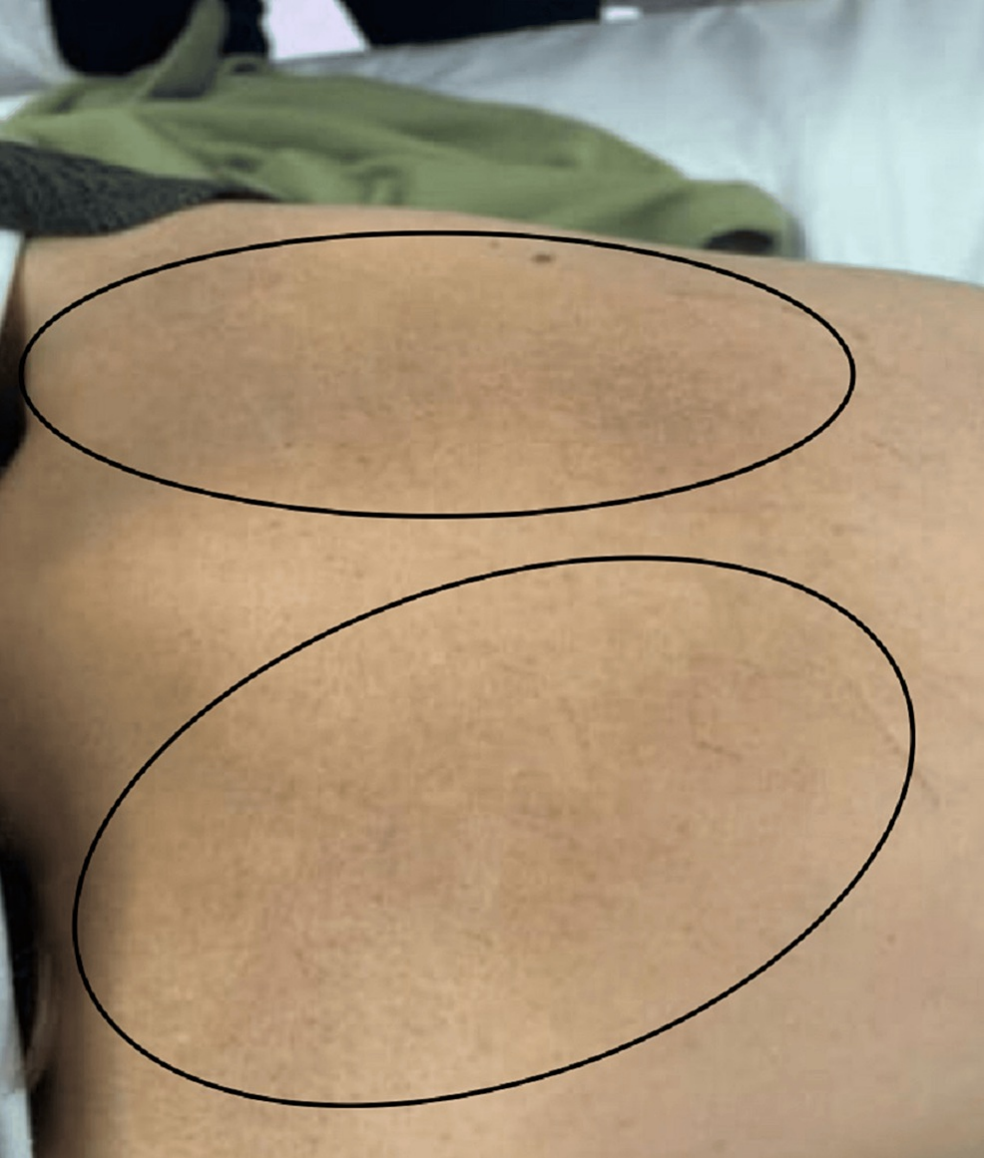
Cutaneous hyperpigmentation on the upper back (bilateral scapular areas within the black oval shapes).

**Figure 3:**
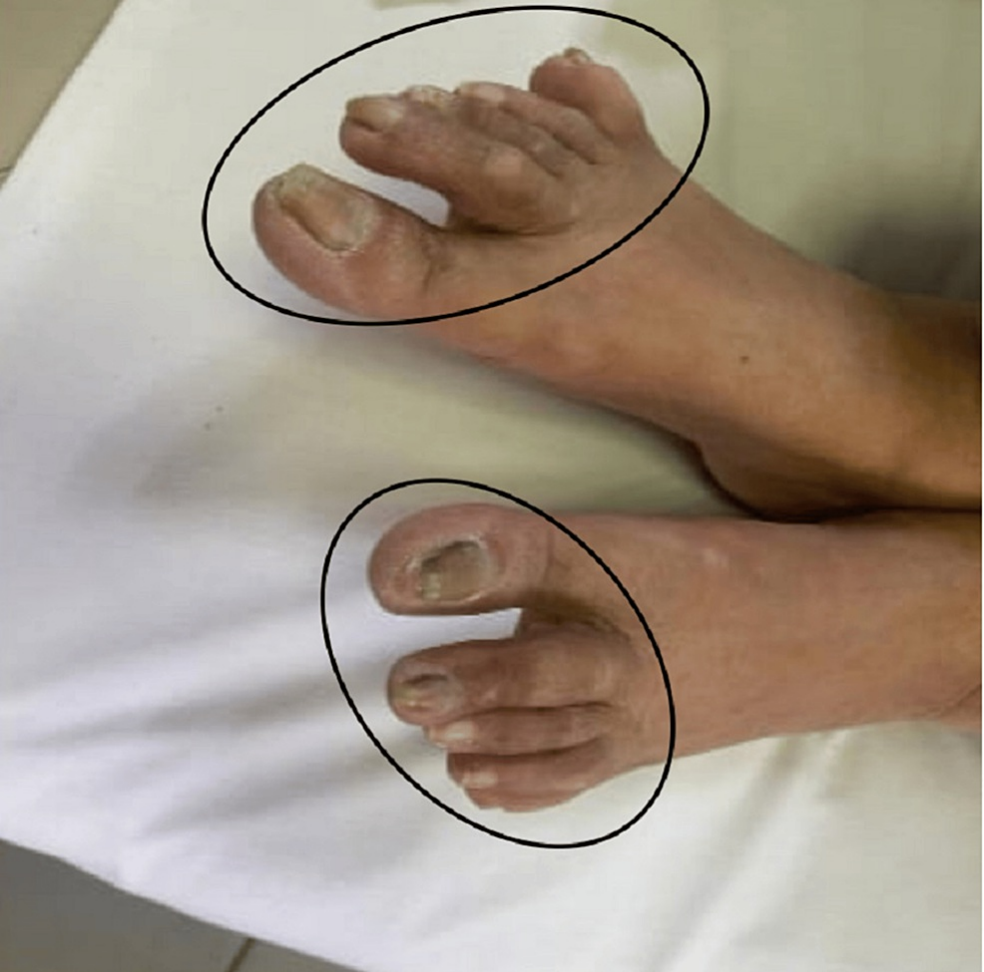
Cutaneous hyperpigmentation on the dorsum of the feet and toes (within the black oval shapes).

She was afebrile, with a pulse rate of 80 beats per minute and a systolic blood pressure of 90 mmHg. Her blood sugar level was elevated at 210 mg/dL, her weight was 86 kg with a body mass index (BMI) of 33.5 kg/m2. Physical examination was completely normal except for mucocutaneous hyperpigmentation at the oral mucosa (Figure 1), skin of the back (Figure 2), and dorsum of the feet (Figure 3).

Initial laboratory investigations at our hospital were relevant for elevated creatinine levels, hyponatremia, hyperkalemia, and microcytic anemia. Relevant laboratory workup is presented in Table 1.

**Table 1:** Relevant laboratory tests.

Test	Patient’s value	Normal range
Creatinine	2 mg/dl	0.4-1 mg/dl
Serum sodium	126 mmol/l	135-145 mmol/l
Serum potassium	6.2 mmol/l	3.7-5.3 mmol/l
Urine osmolality	500 mosmol/l	50-1200 mosmol/l
Urine sodium	89 mmol/l	40-220 mmol/L
White blood cells	6500/microliter	4500-11000/microliter
Hemoglobin	10.1 g/dl	12.1-15.1 g/dl
Mean corpuscular volume	75.6 fl	80-100 fl
Calcium	9.5 mg/dl	8.5-10.5 mg/dl
Magnesium	1.64 mg/dl	1.7-2.6 mg/dl
Phosphorus	3.5 mg/dl	3-4.5 mg/dl

The possible explanation of this patient's hyponatremia with high urine osmolality and urine sodium could be due to hypovolemia secondary to renal salt wasting vs. syndrome of inappropriate antidiuretic hormone secretion (SIADH), this is to be clarified later on.

The patient was admitted to the nephrology service for acute kidney injury (AKI) and hyponatremia. She was started on IV hydration for AKI, free water restriction for hyponatremia, and medical treatment for hyperkalemia, pending further workup. On the next day, creatinine level trended down to 1.3 mg/dL, serum sodium increased to 133 mmol/L, and serum potassium decreased to 5.8 mmol/L.

The findings of hyperpigmentation, hyponatremia, hyperkalemia, and low systolic pressure raised suspicion for primary adrenal insufficiency. Cortisol level (8 a.m.) was ordered and turned out to be very low at <1.5 nmol/L (171-536).

Our patient was diagnosed with primary adrenal insufficiency and was started on intravenous hydrocortisone 50 mg IV every six hours, which normalized her systolic blood pressure, serum sodium, and serum potassium. Given the history of T1DM and subclinical hypothyroidism, combined with primary adrenal insufficiency, our patient was diagnosed with APS-II (Schmidt syndrome).

Her hyponatremia, in the setting of AKI, was likely related to hypovolemia from renal salt wasting secondary to primary adrenal insufficiency.

The hydrocortisone was rapidly tapered by 50 mg each day to 50 mg intravenous every 12 hours, and then she was shifted on the next day to oral hydrocortisone 10 mg daily in the morning and 5 mg daily in the afternoon, which is considered the daily requirement for hydrocortisone or the "physiological dose." She was educated to check her blood pressure and blood sugar initially on a daily basis and then after one week on a weekly basis. The patient was also instructed to adjust the dose of hydrocortisone upwards on days of illnesses and stresses, after discussing the illness or stress with her endocrinologist. The endocrinologist determines the degree of increase according to the severity of illness or stress, to avoid consequent adrenal crisis.

## Discussion

APS-II is the most common group of APS disorders, characterized by the presence of a combination of AI, also known as Addison disease, as its defining component, along with ATD (Hashimoto’s thyroiditis or Graves’ disease) and T1DM [2,3]. Of APS-II patients, 41% have T1DM and ATD association, whereas AI combined with ATD or T1DM accounts for 14.6% and 3.3%, respectively [3].

Several endocrine disorders, such as hypogonadism and hypoparathyroidism, as well as non-endocrine autoimmune disorders, including celiac disease, vitiligo, pernicious anemia, myasthenia gravis, autoimmune gastritis, autoimmune hepatitis, Sjögren syndrome, systemic lupus erythematous, rheumatoid arthritis, and alopecia, are less commonly associated with APS-II [3,4]. Many years to decades may separate the onset of different comorbidities [3]; the time interval between AI and ATD is the shortest whereas it is the longest between T1DM and ATD [5].

The etiology of APS-II is based on genetic and environmental factors, resulting in a prolonged phase of tissue destruction preceding overt APS-II. Its inheritance pattern is complex, with the predominant role of genes on chromosome 6 and a strong association with human leukocyte antigen (HLA) alleles DR3 and B8. Other risk factors are suggested when the concordance in monozygotic twins for this syndrome is less than 100%. Moreover, the cascade of DR3-associated autoimmune disease can be triggered by epigenetic external factors (viral or bacterial infections), and psychosocial and environmental factors (nicotine consumption or hormonal influence), yet exposure to these pathogens does not necessarily lead to abnormalities [2,3].

These patients can have a variety of symptoms, including anorexia, fatigue, nausea, vomiting, generalized weakness, abdominal discomfort, and diarrhea. If AI is diagnosed, patients may exhibit mucosal and cutaneous hyperpigmentation, hypoglycemia, and orthostatic hypotension, and sometimes they can present with adrenal crisis and shock-like features. If T1DM is present, these patients may develop polyuria and polydipsia with hyperglycemia. For hypothyroidism, bradycardia and delayed tendon reflexes can occur [6].

Testing for autoantibodies to 21-hydroxylase, glutamic acid decarboxylase, insulin-associated antigen, cytoplasmic islet cell, thyroperoxidase, TSH-receptor, thyroglobulin, and tissue transglutaminase should be done when APS-II is clinically suspected. This is particularly true when Addison's disease is the initial presentation, but screening for AI is less useful when the original presentation is ATD or T1DM [7]. Antibodies against steroidal enzymes, such as 21-hydroxylase, have a strong prognostic value and can be used to identify people who are at risk of developing AI. This could avoid a delayed diagnosis of adrenal insufficiency [3].

The main treatment for APS-II involves replenishing the deficient hormones, like any other single-organ endocrine disorder. However, it is important to start with adrenal steroid replacement before thyroxine to prevent an Addisonian crisis [8]. Numerous immunosuppressants and immune-modulators have been investigated due to the autoimmune nature of this condition, but none of them are currently used due to side effects [6]. APS-II increases the risk of adrenal crisis 2.5 times in the presence of AI, as well as the risk of developing severe hypothyroidism and diabetic ketoacidosis. Thus, early diagnosis and treatment are crucial to reduce morbidity and mortality [6].

It is advisable to screen for thyroid dysfunction in the first-degree relatives of patients with APS-II due to the high prevalence of unrecognized endocrine disorders, particularly Hashimoto thyroiditis [3].

In our case, the diagnosis of AI was suspected based mainly on hyponatremia due to SIADH. Cortisol deficiency leads to a decrease in systemic blood pressure and cardiac output, causing hypersecretion of antidiuretic hormone (ADH). The latter can also be triggered by salt wasting and hypovolemia due to the aldosterone deficiency seen in primary adrenal insufficiency. In addition, the negative feedback in which cortisol suppresses ADH secretion is interrupted [9]. Twenty years after she was diagnosed with T1DM, our patient was found to have ATD, and then, after a short period of time, she was diagnosed with AI. She had the three components of APS-II.

APS-II is a chronic illness that necessitates patient understanding of the condition, compliance with medications, regular monitoring of blood pressure and blood sugar, and reporting illnesses to the physician.

## Conclusions

We present a case of a female patient known to have T1DM and diagnosed with APS-II after she was admitted for hyponatremia and AKI. APS-II, which is defined by the presence of AI, T1DM, and/or ATD, is a rarely reported condition, especially when the three components of the syndrome are present, as in our patient. This case emphasizes on the value of investigating other autoimmune conditions in a patient known to have an autoimmune disease to prevent multiple possible complications.
